# CDK5 promotes renal tubulointerstitial fibrosis in diabetic nephropathy via ERK1/2/PPARγ pathway

**DOI:** 10.18632/oncotarget.9058

**Published:** 2016-04-27

**Authors:** Xiaoyan Bai, Xiaoyan Hou, Jianwei Tian, Jian Geng, Xiao Li

**Affiliations:** ^1^ Division of Nephrology, Nanfang Hospital, Southern Medical University, National Clinical Research Center for Kidney Disease, State Key Laboratory of Organ Failure Research, Guangdong Provincial Institute of Nephrology, Guangzhou, Guangdong, PR China; ^2^ Division of Nephrology, The First Affiliated Hospital, Inner Mongolia Medical University, Hohhot, Inner Mongolia, PR China; ^3^ Department of Pathology, Nanfang Hospital, Southern Medical University, Guangzhou, Guangdong, PR China; ^4^ Department of Emergency, Nanfang Hospital, Southern Medical University, Guangzhou, Guangdong, PR China

**Keywords:** CDK5, ERK1/2, PPARγ, tubulointerstitial fibrosis, diabetic nephropathy

## Abstract

Cyclin-dependent kinase 5 (CDK5) has been documented in podocyte injuries in diabetic nephropathy (DN), however its role in renal tubular epithelial cells has not been elucidated. We report here that CDK5 is detrimental and promotes tubulointerstitial fibrosis (TIF) via the extracellular signal-regulated kinase 1/2 (ERK1/2)/peroxisome proliferator-activated receptor gamma (PPRAγ) pathway in DN. In high glucose cultured NRK52E cells, blocking CDK5 activity inhibited epithelial-to-mesenchymal transition (EMT) and fibrosis via ERK1/2/PPARγ pathway. In diabetic rats, CDK5 inhibitor roscovitine decreased renal fibrosis and improved renal function as demonstrated by a decrease in levels of blood urine nitrogen (BUN), serum creatinine and β2-microglobulin. Further studies revealed that improved renal fibrosis and function in diabetic rats were associated with inactivation of ERK1/2 and PPARγ signaling pathways. In late staged DN patients, the upregulation of CDK5 and p35 activated phosphorylated ERK1/2 and PPARγ, leading to decreased levels of E-cadherin but increased Vimentin and Collagen IV. Accordingly, renal fibrosis and function were worsened as revealed by decreased estimated glomerular filtration rate (eGFR) and increased serum BUN, creatinine, β2-microglobulin, 24-hour proteinuria and urine albumin to creatinine ratio (UACR). These findings demonstrate a novel mechanism that CDK5 increases tubulointerstitial fibrosis by activating the ERK1/2/PPARγ pathway and EMT in DN. CDK5 might have therapeutic potential in diabetic nephropathy.

## INTRODUCTION

Worldwide, the prevalence of diabetes is growing, yet treatment for the disease is still limited. Despite a gradual decline in mortality from macrovascular complications of type 2 diabetes, diabetic nephropathy (DN) remains a leading cause of end-stage renal disease (ESRD) globally, with its incidence being on a rising trend. Notably, once nephropathy develops, approximately 40% of patients inevitably progress to ESRD. Therefore, biomarkers for early risk stratification in DN need to be identified so that those at higher risk of progression to ESRD are treated promptly at early stages of nephropathy development.

Renal tubulointerstitial fibrosis (TIF) is an important pathway leading to end-stage renal failure. The degree of TIF relates with declined renal function, manifested with tubular atrophy and accumulation of extracellular matrix (ECM) proteins, such as fibronectin and collagens. Epithelial-to-mesenchymal transition (EMT) is considered a major contributor to TIF and enhanced EMT triggers a cascade of signaling pathways leading to deteriorated fibrosis, and ultimately decreased renal function.

Cyclin-dependent kinase 5 (CDK5) is a proline-directed serine/threonine kinase that belongs to the family of CDKs, which play essential roles in cell cycle regulation. CDK5 is highly conserved in mammals and is expressed in all tissues, with the highest expression levels being observed in the central nervous system [1, 2]. Recent studies have shown that CDK5 modulates cell maturation, differentiation, migration and apoptosis in various tissues. In the kidney, CDK5 has been documented to form cyclin I/CDK5 or p35/CDK5 complexes that protect podocytes against apoptosis [3]. CDK5 expression has also been identified in renal tubular epithelial cells and CDK5 inhibition by classical CDK inhibitors, such as roscovitine, induces cell recovery by promoting the formation of pro-survival CDK5/cyclin I complexes [4]. High glucose has been demonstrated to increase the expression of CDK5 in cultured podocytes and knockdown of CDK5 attenuated podocyte apoptosis induced by high glucose stimulation [5]. These findings suggest the involvement of CDK5 in podocyte injuries in DN. However, unlike in podocytes, CDK5 does not affect apoptosis in renal tubular epithelial cells [4]. Accordingly, whether and how CDK5 regulates renal tubular epithelial cells needs further investigation.

Roscovitine, a CDK5 inhibitor, has been reported to decrease matrix protein transcription and fibrosis independent of cell cycle regulation [6]. CDK5 function is both necessary and sufficient in cultured adipocytes to phosphorylate peroxisome proliferator-activated receptor gamma (PPARγ) [7]. Previous studies have revealed that CDK5 modification of PPARγ could be a major source of gene dysregulation and pathology of adipose tissues in obesity [6]. Under diabetic conditions, extracellular signal-regulated kinase 1/2 (ERK1/2) activation has been shown to stimulate PPARγ signaling through regulating CDK5 activity [8]. The finding that PPARγ ameliorates EMT and thus alleviates TIF has also been shown in multiple studies [9, 10]. However, whether CDK5 promotes ERK1/2/PPARγ–mediated EMT and TIF in DN and the underlying mechanisms remain unknown.

In view of the interplay between CDK5, ERK1/2, and PPARγ, we hypothesize that CDK5 acts as a critical factor in the ERK1/2-PPARγ axis to activate the cascade of reactions leading to EMT and renal TIF in DN. Mechanisms of PPARγ-induced downstream cellular events and ERK1/2-mediated stimulation of PPARγ by CDK5 were also investigated.

## RESULTS

### High glucose stimulates the expression of CDK5 and p35 and elevates CDK5 kinase activity in NRK-52E cells

**Figure 1:**
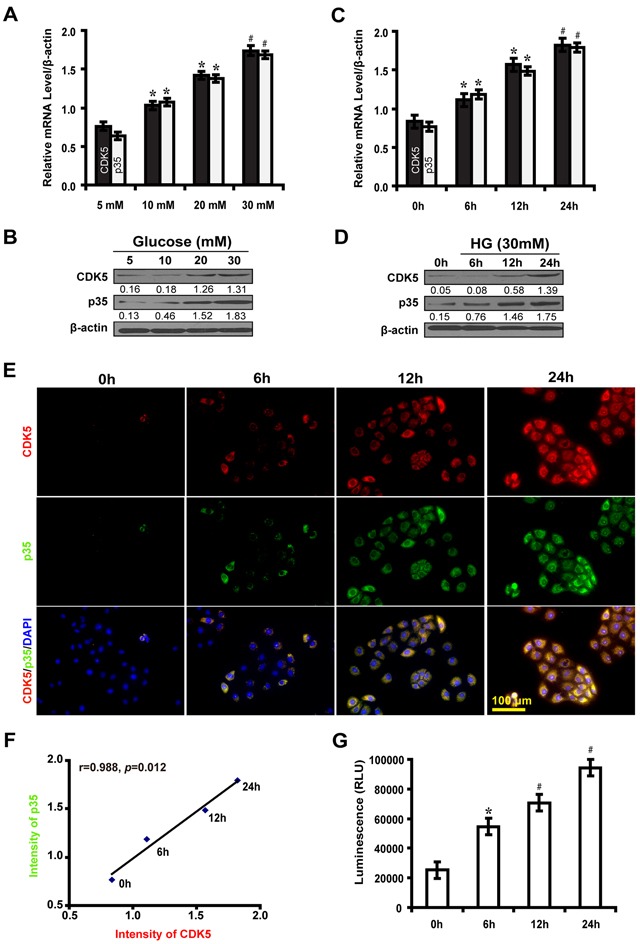
High glucose induces the expression of CDK5 and p35 and elevates CDK5 kinase activity in NRK-52E cells **A, B.** Increased expression levels of CDK5 and p35 at indicated concentrations of glucose compared with the control (5 mM). **C, D.** Increased expression levels of CDK5 and p35 at indicated time points in high glucose medium (30 mM) compared with the control (0 h). **E.** Increased expression levels of CDK5 (red) and p35 (green) at indicated time points. **F.** Positive correlation between the expression of CDK5 and p35 at indicated time points. **G.** Increased CDK5 kinase activity in a time-dependent manner compared with the control (0 h). Results are presented as mean ± SD of three independent experiments. *P<0.05, #*P*<0.001. 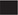, CDK5; 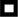, p35.

To investigate the effect of high glucose on the expression of CDK5 and p35, NRK-52E cells were cultured in different concentrations of glucose (5, 10, 20 and 30 mM) for 12 hours. The expression level of CDK5 and p35 increased in a dose-dependent manner (Figure 1A and 1B). Then, we examined the time course expression of CDK5 and p35 in high glucose condition (30 mM). We found that the expression level of CDK5 and p35 increased at different time points (0, 6, 12 and 24h) in high glucose medium (Figure 1C and 1D). As illustrated in immunofluorescence microscopy, the expression of CDK5 and p35 were positively correlated in a time course (Figure 1E and 1F). Furthermore, CDK5 kinase activity revealed that high glucose-treated NRK52E cells exhibited an increased CDK5 kinase activity in a time-dependent manner compared with the control (Figure 1G). Taken together, these results indicate that high glucose stimulates the expression of CDK5 kinase activity.

### CDK5 inhibition decreases phosphorylation of ERK1/2 and PPARγ in high glucose cultured NRK-52E cells

**Figure 2:**
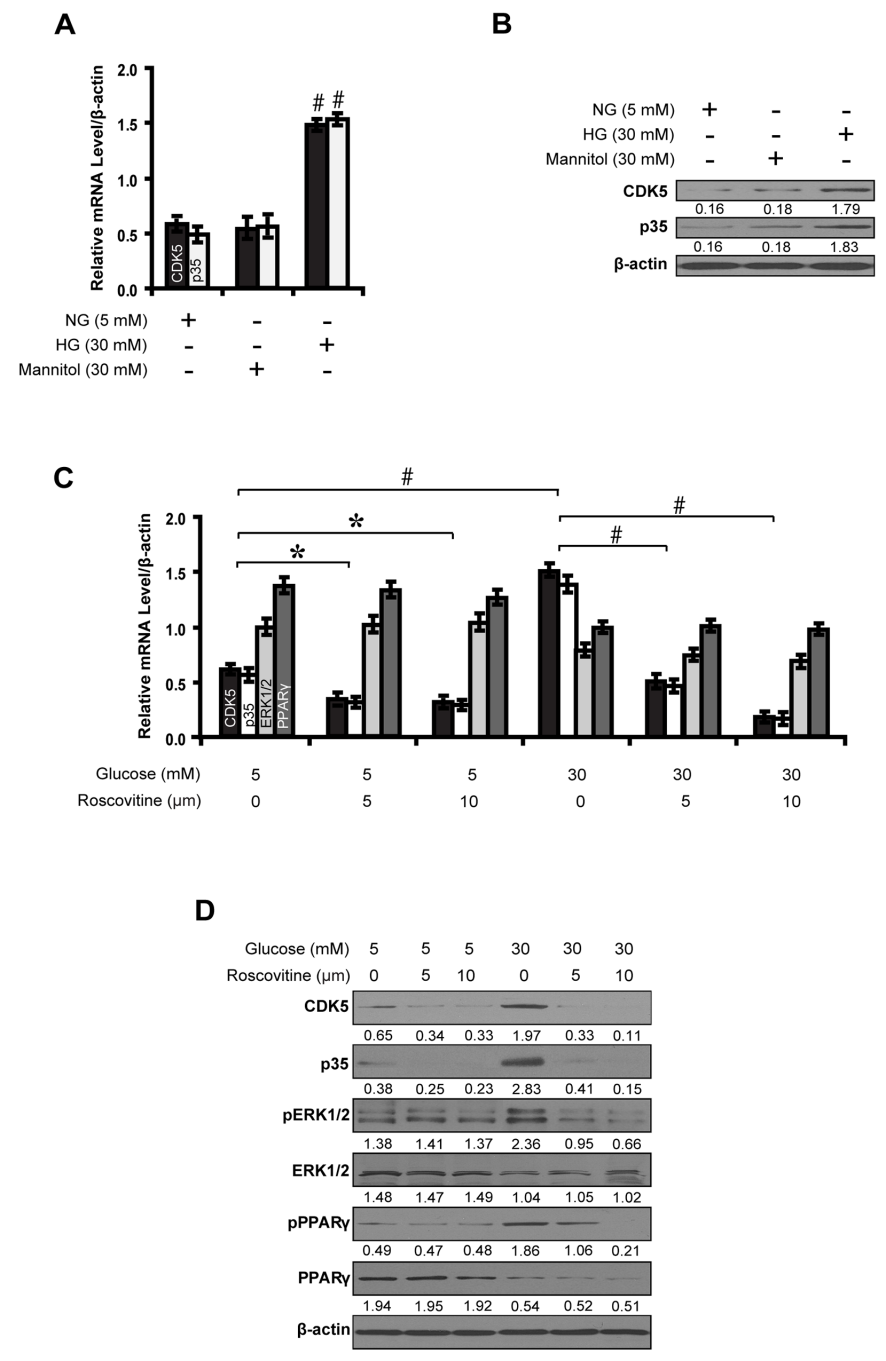
Roscovitine decreases phosphorylated ERK1/2 and PPARγ in high glucose cultured NRK-52E cells **A, B.** Expression levels of CDK5 and p35 compared between groups. **C, D.** Roscovitine decreased phosphorylated ERK1/2 and PPARγ in high glucose condition. Results are presented as mean ± SD of three independent experiments. *P<0.05, #*P*<0.001. NG, normal glucose; HG, high glucose. 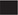, CDK5; 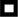, p35; 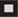, ERK1/2; 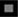, PPARγ.

Given the increased activity of CDK5 with increasing concentrations of glucose, we next investigated the potential effects of high glucose and CDK5 inhibition on the activity of ERK1/2 and PPARγ. First, there was an increase in the level of CDK5 (mRNA: 1.09-fold; protein: 1.63-fold) and p35 (mRNA: 1.13-fold; protein: 1.67-fold) in 30 mM high glucose condition compared with normal glucose. Mannitol (30 mM) had no effect on the expression of these two molecules (Figure 2A and 2B). Next, we found that high glucose (30 mM) increased phosphorylated ERK1/2 and PPARγ by 0.98- and 1.37-fold, respectively, compared with controls. But roscovitine decreased phosphorylated ERK1/2 and PPARγ in a dose-dependent manner (Figure 2C and 2D).

### PPARγ inhibits EMT in high glucose cultured NRK-52E cells

**Figure 3:**
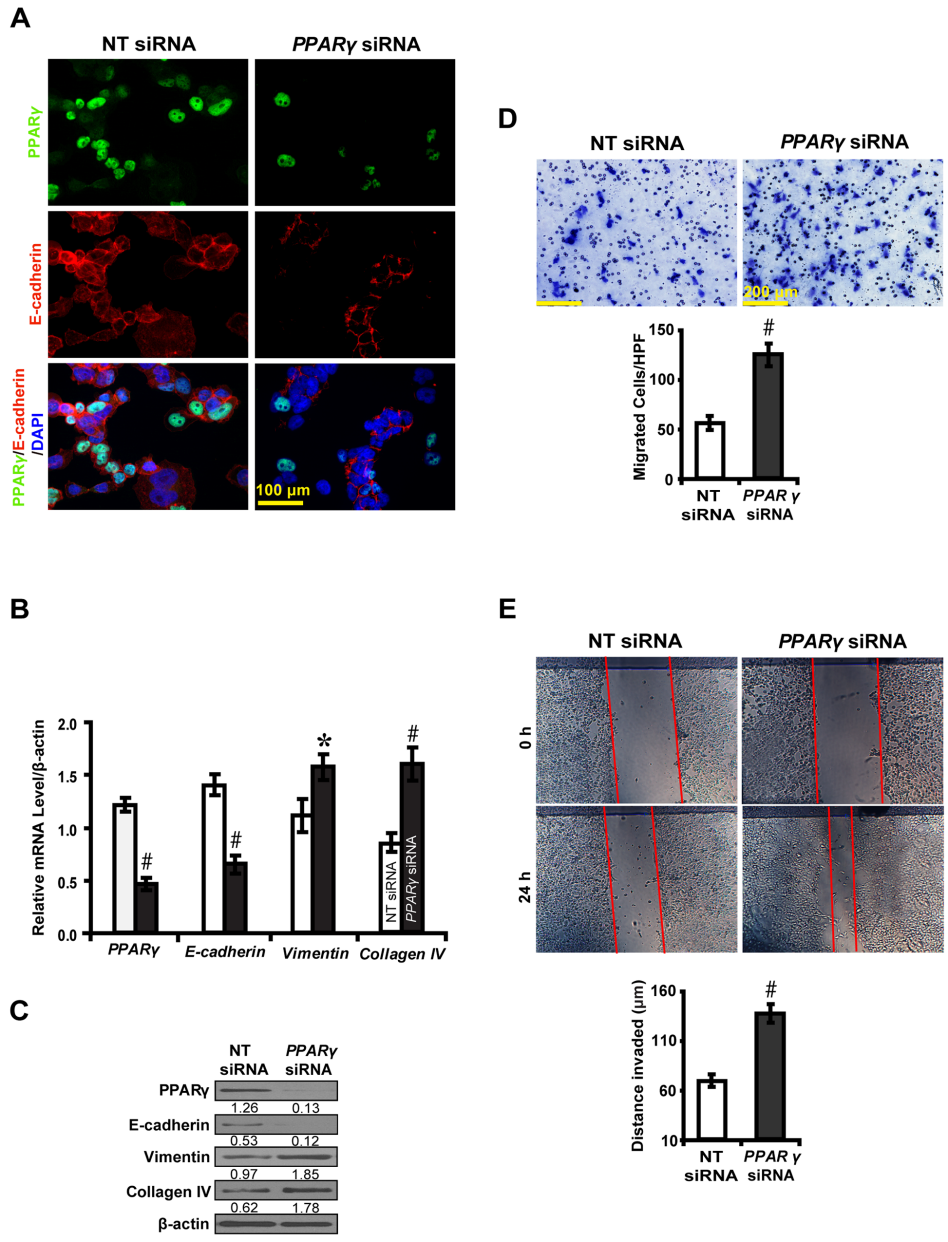
PPARγ siRNA promotes EMT in high glucose cultured NRK-52E cells **A.** Downregulated E-cadherin but **B, C.** upregulated Vimentin and Collagen IV by *PPARγ* siRNA. **D.** Increased migrated cells by *PPARγ* siRNA. **E.** Increased invaded distance by *PPARγ* siRNA. Results are presented as mean ± SD of three independent experiments. *P<0.05, #*P*<0.001 compared with NT siRNA. 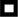, NT siRNA; 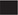, *PPARγ* siRNA.

**Figure 4:**
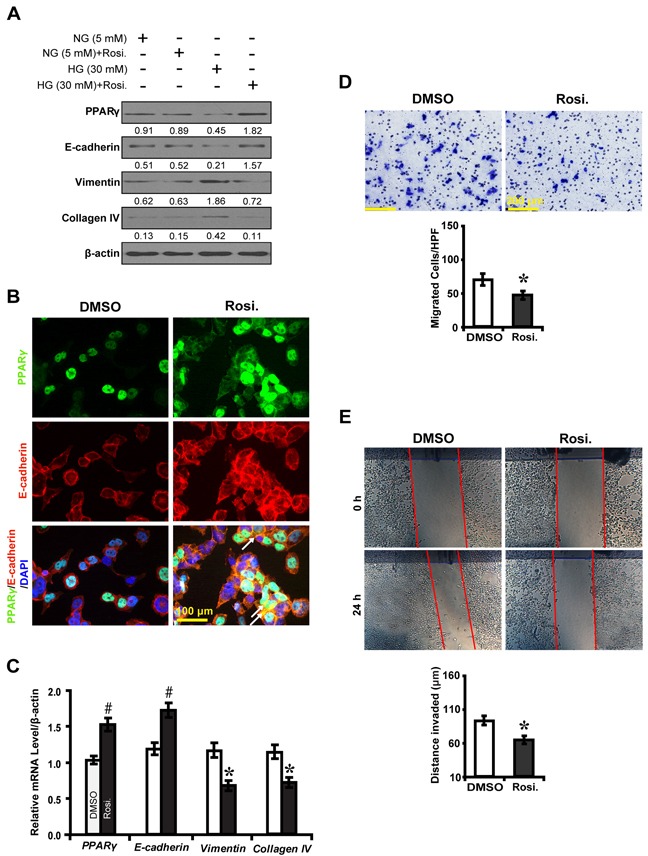
PPARγ agonist rosiglitazone inhibits EMT in high glucose cultured NRK-52E cells **A.** Effect of rosiglitazone on the expression of E-cadherin, Vimentin and Collagen IV compared between conditions. **B.** Increased PPARγ (green) and E-cadherin (red) with rosiglitazone treatment and overlaid image showing colocalization (yellow, arrows) in high glucose condition (30 mM). **C.** Rosiglitazone upregulated E-cadherin but downregulated Vimentin and Collagen IV. **D.** Decreased migrated cells with rosiglitazone treatment. **E.** Decreased invaded distance with rosiglitazone treatment. Results are presented as mean ± SD of three independent experiments. *P<0.05, #*P*<0.001 compared with DMSO. EMT, epithelial-to-mesenchymal transition; Rosi.: rosiglitazone. 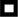, DMSO; 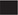, Rosi.

To further decipher whether PPARγ inhibits EMT, we knocked down the expression of PPARγ in high glucose cultured NRK-52E cells and examined downstream gene expressions and biological features of the cells. PPARγ siRNA led to decreased E-cadherin expression, but increased Vimentin and Collagen IV (Figure 3A, 3B and 3C). Notably, PPARγ silencing promoted NKR-52E cells to migrate (Figure 3D) and invade (Figure 3E). In contrast, rosiglitazone upregulated the level of E-cadherin, but downregulated Vimentin and Collagen IV in high glucose condition instead of normal glucose condition (Figure 4A, 4B and 4C). Immunofluorescece microscopy also revealed that rosiglitazone stimulated the nuclear to cytoplasmic translocation of PPARγ and co-localized with E-cadherin (Figure 4B). Furthermore, rosiglitazone inhibited the cells to migrate (Figure 4D) and invade (Figure 4E). These data collectively suggest PPARγ suppresses EMT in hyperglycemic conditions *in vitro*.

### ERK1/2 inhibition impedes PPARγ-mediated EMT in high glucose cultured NRK-52E cells

**Figure 5:**
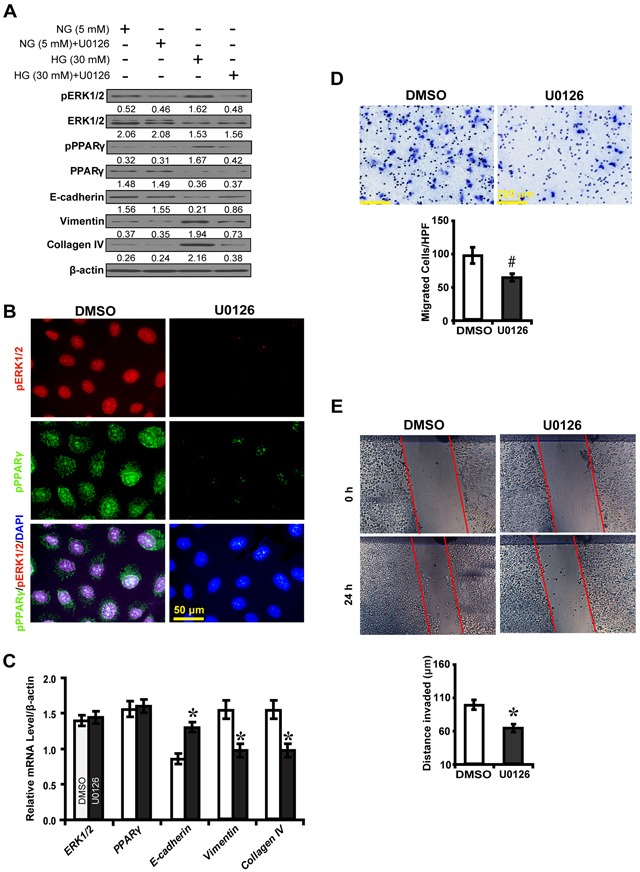
U0126 inhibits PPARγ-mediated EMT in high glucose cultured NRK-52E cells **A.** Effect of U0126 on the expression of phosphorylated ERK1/2 and PPARγ, E-cadherin, Vimentin and Collagen IV compared between conditions. **B.** Decreased phosphorylated ERK1/2 (red) and phosphorylated PPARγ (green) with U0126 treatment in high glucose condition (30 mM). **C.** Increased E-cadherin but decreased Vimentin and Collagen IV expression with U0126 treatment. **D.** Decreased migrated cells with U0126 treatment. **E.** Decreased invaded distance with U0126 treatment. Results are presented as mean ± SD of three independent experiments. *P<0.05, #*P*<0.001 compared with DMSO. 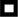, DMSO; 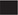, U0126.

To further explore the effect of ERK1/2 inhibition on downstream gene expression and EMT, an ERK1/2 inhibitor U0126 was used to treat high glucose cultured NRK52E cells. As was shown in Figure 5A, U0126 significantly decreased phosphorylated ERK1/2 and PPARγ, Vimentin and Collagen IV, but increased E-cadherin in high glucose condition as compared to normal glucose condition. U0126 decreased phosphorylated PPARγ, with upregulation of E-cadherin but downregulation of Vimentin and Collagen IV (Figure 5B and 5C). U0126 inhibited high glucose cultured NRK52E cells to migrate (Figure 5D) and invade (Figure 5E). These data suggest ERK1/2 inhibition impedes PPARγ-mediated EMT process in high glucose cultured NRK-52E cells *in vitro*.

### Inhibition of CDK5 dampens EMT via ERK1/2/PPARγ pathway in high glucose cultured NRK-52E cells

**Figure 6:**
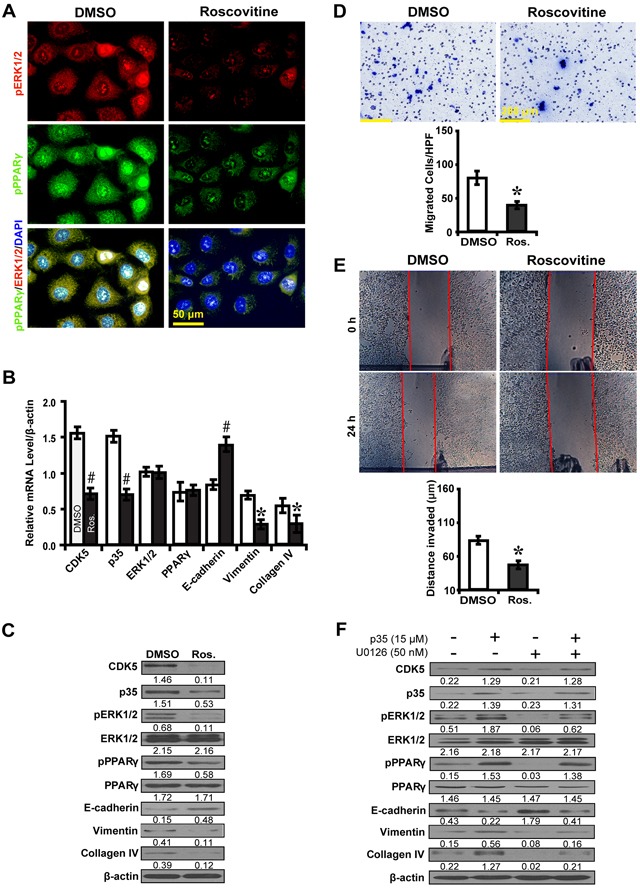
Roscovitine inhibits EMT via ERK1/2/PPARγ pathway in high glucose cultured NRK-52E cells **A.** Decreased phosphorylated ERK1/2 (red) and PPARγ (green) with roscovitine treatment. **B, C.** Roscovitine downregulated phosphorylated ERK1/2 and PPARγ with increased E-cadherin but decreased Vimentin and Collagen IV. **D.** Decreased migrated cells with roscovitine. **E.** Decreased invaded distance with roscovitine. **F.** P35 attenuates the inhibitory effect of U0126 on the expression of phosphorylated ERK1/2 and PPARγ, E-cadherin, Vimentin and Collagen IV. Results are presented as mean ± SD of three independent experiments. *P<0.05, #*P*<0.001 compared with DMSO. Ros., Roscovitine. 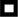, DMSO; 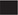, Ros.

**Figure 7:**
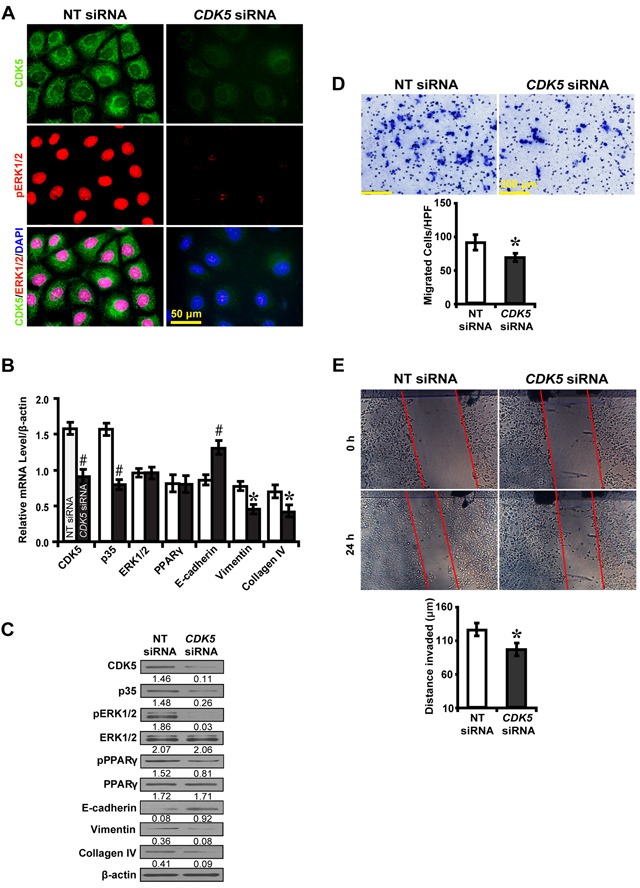
*CDK5* siRNA inhibits EMT via ERK1/2/PPARγ pathway in high glucose cultured NRK-52E cells **A.** Decreased CDK5 (green) and phosphorylated ERK1/2 (red) with *CDK5* siRNA. **B, C.** *CDK5* siRNA increased E-cadherin but decreased phosphorylated ERK1/2 and PPARγ, Vimentin and Collagen IV. **D.** Decreased migrated cells with *CDK5* siRNA. **E.** Decreased invaded distance with *CDK5* siRNA. Results are presented as mean ± SD of three independent experiments. *P<0.05, #*P*<0.001 compared with NT siRNA. EMT, epithelial-to-mesenchymal transition. 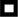, NT siRNA; 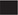, *CDK* siRNA.

To further explore whether CDK5 inhibition inactivates ERK1/2/PPARγ and the EMT process, roscovitine was used to treat high glucose cultured NRK52E cells. Downstream gene expressions and biological features were investigated. Immunofluorescence microscopy revealed that roscovitine decreased phosphorylated ERK1/2 and PPARγ (Figure 6A). Correspondingly, roscovitine decreased the level of CDK5 and p35 with upregulation of E-cadherin, but downregulation of Vimentin and Collagen IV (Figure 6B and 6C). Moreover, roscovitine inhibited the ability of high glucose cultured NRK52E cells to migrate (Figure 6D) and invade (Figure 6E). Next, we treated NRK52E cells with U0126 and p35 to further elucidate whether the effect of CDK5 depends on ERK1/2. CDK5 activation attenuated the inhibitory effect of U0126 on the expression of phosphorylated ERK1/2 and PPARγ and downstream EMT markers (Figure 6F). Knocking down CDK5 with siRNA had similar effects (Figure 7). These data suggest ERK1/2/PPARγ signaling as the downstream pathway of CDK5 in promoting EMT in NRK-52E cells *in vitro*.

### Inhibition of CDK5 kinase activity ameliorates tubulointerstitial fibrosis in diabetic rats

**Figure 8:**
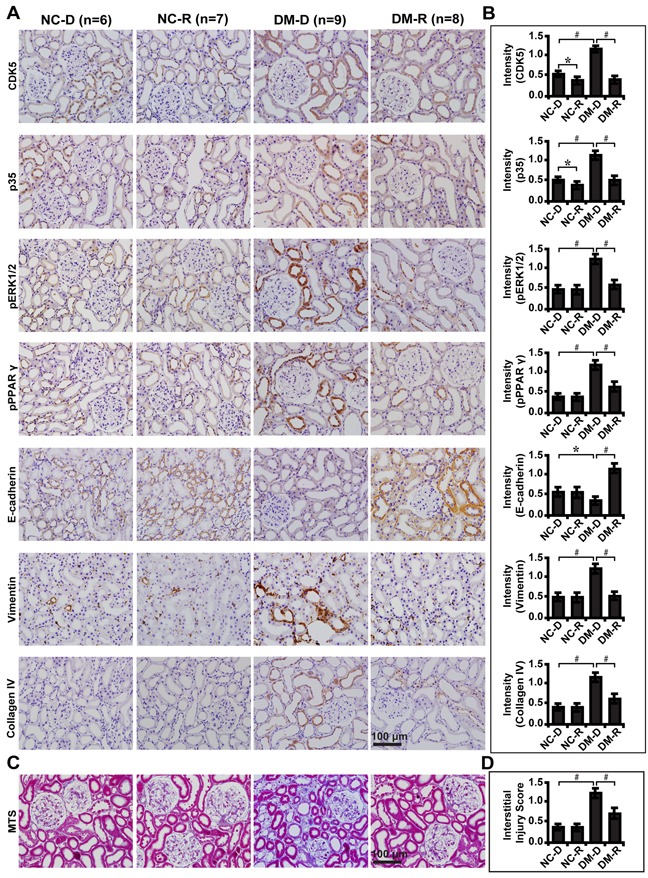
Effect of roscovitine on diabetic rats and controls **A.** Immunohistochemical staining of CDK5, p35, phosphorylated ERK and PPARγ, E-cadherin, Vimentin and Collagen IV indiabetic rats and controls with or without roscovitine treatment. **B.** Quantification of the staining intensity compared between groups. **C.** Representative images of MTS in diabetic rats and controls and **D.** interstitial injury score compared between groups. Results are presented as mean ± SD for groups of 6-9 rats of three independent experiments. *P<0.05, #*P*<0.001. NC-D, normal control rats treated with dimethyl sulphoxide; NC-R, normal control rats treated with roscovitine; DM-D, diabetic rats treated with dimethyl sulphoxide; DM-R, diabetic rats treated with roscovitine; MTS, Masson's trichrome stain.

**Figure 9:**
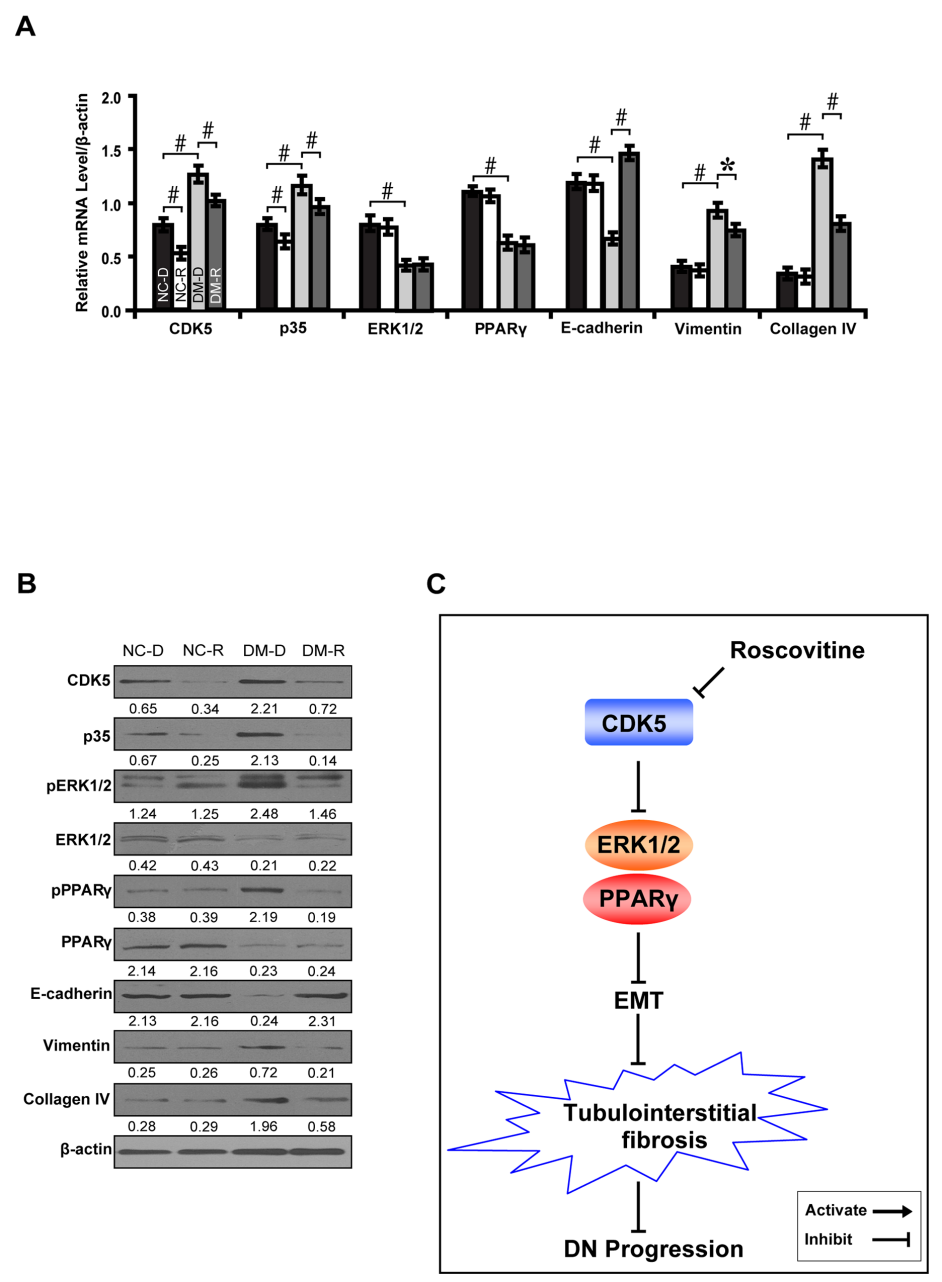
Roscovitine inhibits EMT and fibrosis in diabetic rats **A, B.** Expression levels of CDK5, p35, phosphorylated ERK and PPARγ, E-cadherin, Vimentin and Collagen IV in microdissected renal tubules compared between groups. **C.** A schematic illustration showing that roscovitine inhibited DN progression through dampening the EMT process and fibrosis via the ERK1/2/PPARγ pathway. Results are presented as mean ± SD for groups of 6-9 rats of three independent experiments. *P<0.05, #*P*<0.001. NC-D, normal control rats treated with dimethyl sulphoxide; NC-R, normal control rats treated with roscovitine; DM-D, diabetic rats treated with dimethyl sulphoxide; DM-R, diabetic rats treated with roscovitine; EMT, epithelial-to-mesenchymal transition; DN, diabetic nephropathy. 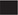, NC-D; 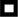, NC-R; 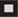, DM-D; 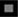, DM-R.

To further demonstrate whether CDK5 inhibition decreases tubulointerstitial fibrosis through the ERK1/2/PPARγ pathway *in vivo*, we treated streptozotocin-induced diabetic rats with roscovitine and renal function was examined. Compared with normal controls, roscovitine downregulated phosphorylated ERK1/2 and PPARγ with concomitant increase in E-cadherin, but decrease in Vimentin and Collagen IV (Figure 8A and 8B). Correspondingly, roscovitine decreased renal tubulointerstitial fibrosis of diabetic rats (Figure 8C and 8D). Similar results were obtained in microdissected renal tubules (Figure 9A and 9B). Furthermore, we noted with interest that roscovitine improved renal function as demonstrated by a decrease in the level of serum BUN, creatinine and β2-microglobulin in diabetic rats (Table 1). These data collectively suggest that roscovitine was effective in decreasing tubulointerstitial fibrosis via the ERK1/2/PPARγ pathway in diabetic rats (Figure 9C).

**Table 1:** Parameters for diabetic rats treated with roscovitine at week 12

Variables	DMSO (n=9)	Roscovitine (n=8)
Serum BUN (mmol/L)	7.51±0.48	6.84±0.67*
Scr (umol/L)	115.32±1.27	106.46±2.11*
Serum β2-microglobulin (mcg/mL)	0.11±0.07	0.06±0.03#
Blood glucose (mmol/L)	23.15±2.15	23.37±0.74
Body weight (g)	183.24±2.45	183.91±1.48
Kidney weight (g)	1.81±0.12	1.79±0.16
Kidney/body weight (%)	1.04	1.05

DMSO, dimethyl sulphoxide; Serum BUN, serum blood urea nitrogen; Scr, serum creatinine;

* *P*<0.01;

# *P*<0.001.

### CDK5 and downstream signalings reflect tubulointerstitial fibrosis in renal biopsies of DN patients

**Figure 10:**
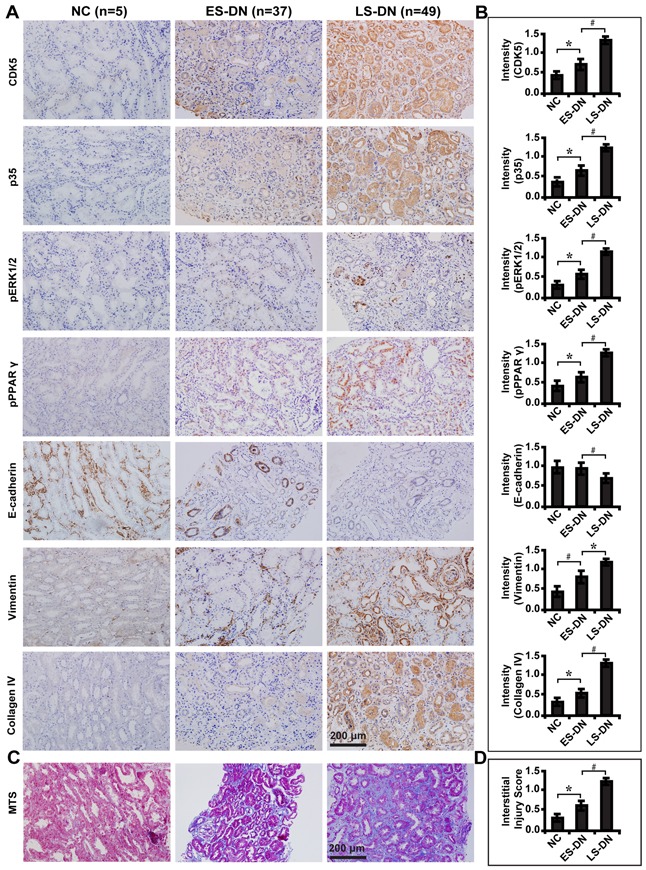
Expression levels of CDK5 and related markers in renal biopsies of DN patients and relationship with renal fibrosis **A.** Immunohistochemical staining of CDK5, p35, phosphorylated ERK1/2 and PPARγ, E-cadherin, Vimentin and Collagen IV in ES-DN (n=49), LS-DN (n=37) and NC (n=5). **B.** Quantification of the staining intensity compared between groups. **C.** Deteriorated TIF in LS-DN compared with ES-DN and NC by MTS and **D.** interstitial injury score compared between groups. Results are presented as mean ± SD of three independent experiments. *P<0.05, #*P*<0.001. NC, normal controls; ES-DN, early staged diabetic nephropathy; LS-DN, late staged diabetic nephropathy; TIF, tubulointersitial fibrosis; MTS, Masson's trichrome stain.

Next, we examined the expression of these molecules in renal biopsy samples of DN patients. Immunohistochemical staining (Figure 10A) and quantification of the staining intensity (Figure 10B) revealed that CDK5 and p35 were significantly elevated in late staged DN compared with the early stage and normal controls. There was a corresponding increase in the expression level of phosphorylated ERK1/2 and PPARγ, Vimentin and Collagen IV, but decreased E-cadherin. Compared with early staged DN, renal fibrosis was worsened (Figure 10C and 10D) and renal function dropped as demonstrated by increased level of serum BUN, creatinine, β2-microglobulin, 24 hour proteinuria and UACR and decreased estimated glomerular filtration rate (eGFR) (Table 2). There was no difference in the gender, but an increase in the age and history of diabetes mellitus in late staged DN patients (Table 2). These results indicate that CDK5 could be used as a marker to reflect the severity of tubulointerstitial fibrosis and renal function in diabetic nephropathy.

**Table 2:** Biological parameters for early and late staged DN patients

Variables	Early Stage (n=37)	Late Stage (n=49)
Gender		
Male	18	23
Female	19	26
Age (year)	48.67±8.72	55.53±8.99#
History of DM (year)	7.31±2.86	16.14±3.43#
SBP (mmHg)	154.26±23.48	158.43±21.37
DBP (mmHg)	85.26±5.43	86.39±5.16
Blood glucose (mmol/L)	9.12±1.28	9.11±1.19
Serum BUN (mmol/L)	8.78±3.14	9.81±3.16*
Scr (umol/L)	124.36±3.37	143.62±5.32#
Serum β2-microglobulin (mcg/mL)	5.31±1.37	6.07±1.16*
Proteinuria (g/24h)	0.46±0.28	5.18±2.26#
UACR (mg/mmol)	186.75±11.13	315.42±16.34#
eGFR (ml/min.1.73m^2^)	83.60±7.73	40.40±7.11#

DM, diabetes mellitus; eGFR, estimated glomerular filtration rate; SBP, systolic blood pressure; DBP, diastolic blood pressure; Serum BUN, serum blood urea nitrogen; Scr, serum creatinine; UACR, urine albumin to creatinine ratio;

* *P*<0.05

# *P*<0.001.

## DISCUSSION

The present study indicates a significant role of CDK5 in promoting renal tubulointerstitial fibrosis through the ERK1/2-PPARγ axis in DN.

We demonstrated that high glucose rather than mannitol significantly increased the level of CDK5 and p35 in NRK-52E cells, suggesting that high glucose stimulated the production of CDK5 and p35 independent of the osmotic pressure. We also found that inhibition of CDK5 decreased phosphorylated ERK1/2 and PPARγ and suppressed the EMT process. The observation that ERK1/2 stimulated the expression of PPARγ and downstream EMT markers supports the critical role of ERK1/2 as a pivotal activator in PPARγ-induced EMT and renal tubulointerstitial fibrosis. Additionally, CDK5 inhibition caused NRK52E cells to lose mesenchymal specific features and the tendency to migrate or invade. Moreover, we provide evidences that CDK5 was activated under the high glucose microenvironment and the CDK5/p35 complex interacted and positively correlated with each other. Taken together, the current data suggest CDK5 increases renal tubulointerstitial fibrosis in DN by promoting EMT through the ERK1/2/PPARγ signaling.

The present study also revealed that in diabetic rats, CDK5 inhibitor roscovitine significantly decreased phosphorylated ERK1/2 and PPARγ with attenuated tubulointerstitial fibrosis and improved renal function. In contrast, in DN patients, increased levels of CDK5 and p35 in late staged DN were concomitant with worsened tubulointerstitial fibrosis and renal function. Furthermore, increased phosphorylated ERK1/2 and PPARγ were detected in late staged DN, with decreased E-cadherin, but increased Vimentin and Collagen IV. These results further support the notion that CDK5 inhibition decreases tubulointerstitial fibrosis through suppression of ERK1/2/PPARγ-mediated EMT.

Distinct from other members of the CDK family, the activation of CDK5 does not require binding to cyclins, but rather association with its regulator to perform kinase activity [11]. One major co-activator for CDK5 is p35, and the CDK5/p35 complex has been demonstrated to regulate podocyte differentiation, proliferation and morphology [12]. Surprisingly, the silencing of CDK5 did not affect apoptosis in tubular renal cells, which occurs in other cellular damage models in neural cells or podocytes [13, 14]. Studies have shown that CDK5 can alter pain-related neuronal plasticity by regulating ERK1/2 activation in anesthetized rats [15]. CDK5 has also been characterized to phosphorylate the ERK-activating protein kinase MEK1 in the brain [16]. The present results demonstrated that CDK5 promoted renal tubulointerstitial fibrosis through enhancing phosphorylated ERK1/2 and PPARγ in diabetic nephropathy. We also found that inhibiting CDK5 activity decreased phosphorylated ERK1/2 and PPARγ in high glucose cultured NRK-52E cells. This is in line with other studies showing that PPARγ agonist ameliorates high glucose-induced EMT in renal proximal tubule cells [10].

PPARγ, a ligand-activated transcription factor and member of the nuclear receptor superfamily [17], plays a crucial role in lipid metabolism, epithelial cell differentiation [18] and maintenance of energetic homeostasis [19]. Studies have shown that hypoxia downregulates PPARγ via ERK1/2-dependent mechanism in human pulmonary artery smooth muscle cells [20]. ERK1/2/PPARγ signaling has been found to reduce fat deposits in high fat-fed rats [21] and ERK1/2 activation can induce phosphorylation of PPARγ in adipocytes of obese rodents and humans [22]. Regarded to play a protective role for the organ, PPARγ has also been detected in the inner medullary collecting ducts, proximal tubules and thick ascending limb of Henle's loop of the kidney. PPARγ ligand rosiglitazone has been successfully used as insulin-sensitizing drugs in patients with type 2 diabetes mellitus and exerts beneficial effects on the kidney to ameliorate proteinuria and inflammation [23, 24]. Our data that inhibition of the ERK1/2 signaling decreased phosphorylated PPARγ provide novel evidences demonstrating the correlation between ERK1/2 and PPARγ in tubulointerstitial fibrosis in DN.

The current data also revealed that PPARγ agonist rosiglitazone stimulated the co-localization between PPARγ and E-cadherin, suggesting that the two molecules act together in triggering the EMT process. Previous studies have reported that the PPARγ pathway plays an important role in transforming growth factor-β1-induced EMT in renal tubular epithelial cells [9]. PPARγ has also been identified as an initiator in mediating EMT in colorectal cancer [25]. The finding that PPARγ inhibits the EMT process in the current study further strengthens this notion. Emerging evidences indicate that renal tubular EMT is an important event in initiating tubulointerstitial fibrosis and contributes to disease progression and declined renal function in diabetes. The present data show that inhibiting CDK5 activity attenuated tubulointerstitial fibrosis and improved renal function of diabetic rats. It is conceivable that if the surrounding pathogenic factors are removed timely, the process of EMT has potential possibility to be reversed, hence delaying the onset of end-stage kidney disease.

In conclusion, our study demonstrates a novel mechanism that reducing CDK5 activity inhibits tubulointerstitial fibrosis by blocking ERK1/2/PPARγ-mediated EMT in diabetic nephropathy. CDK5 might have therapeutic potential for diabetic nephropathy.

## MATERIALS AND METHODS

### Cell culture studies

Rat kidney tubular epithelial cells (NRK52E; American Type Culture Collection, Rockville, MD) were maintained in Dulbecco's Modified Eagle's medium (DMEM)/F-12 containing 10% fetal bovine serum (FBS), penicillin (200 U/ml), and streptomycin (200 μg/ml) (Gibco BRL, Grand Island, NY). Cells were cultured in normal glucose (5 mM) for one week, grown to 80%-90% confluence and made quiescent by incubation overnight in a serum-free medium before being exposed to experimental conditions. In the glucose-stimulated group, different concentrations of glucose were added to the culture media at 10 mM, 20 mM, 30 mM, respectively. Osmotic control was performed replacing 30 mM glucose with 30 mM mannitol.

### Immunoprecipitation and CDK5 kinase activity assay

The treated cells were lyzed in 10 mM N-2-hydroxyethylpiperazine-N-ethane-sulphonicacid (HEPES) (pH 7.4), containing 150 mM NaCl, 1% Triton X-100, 0.5% sodium deoxycholate, 1 mM dithiothreitol, 1 mM phenylmethyl sulfonyl fluoride, and complete protease inhibitor mixture. The lysates were centrifuged at 12,000 rpm for 15 minutes. For CDK5 immunoprecipitation, the supernatant was incubated with 50 μl protein A agarose (Santa Cruz Biotechnology, Santa Cruz, CA) and 2 μg anti-CDK5 antibody for 4 hours at 4°C. The immune complex was separated by centrifugation at 4000 rpm. Kinase assay was performed with an ADP-GloTM kinase assay kit (Promega, Madison, WI) according to the manufacturer's protocol. Briefly, immunoprecipitates were washed three times with lysis buffer and then once with reaction buffer and kinase assays were performed in the same buffer by addition of 250 μM ATP and 5 μg substrate histone H1. The mixture was incubated at room temperature for 10 min before 25 μl ADP-GloTM reagents were added to terminate the reaction and deplete the remaining ATP. The results were recorded by measuring the luminescence with a plate-reading luminometer after 50 μl kinase detection reagents were added into the mixture to incubate for 30 minutes. Data are shown as relative light units (RLU) that directly correlate to the amount of ADP produced.

### Drug treatment

CDK5 inhibitor roscovitine (Ros.; 10 μM; R7772; Sigma-Aldrich, St. Louis., MO) and activator p35 (15 μM, Dharmacon, Lafayette, CO), PPARγ agonist rosiglitazone (Rosi.; 50 nM; R2408; Sigma-Aldrich, St. Louis., MO), and ERK1/2 inhibitor U0126 (50 nM; U120; Sigma-Aldrich, St. Louis., MO) were used to treat NRK52E cells. Cells in each group were treated for 72 hours and then harvested for further analyses. Dimethyl sulphoxide (DMSO) was used as a control.

### Transfection of small interfering RNA

Exponential growth phase cells were plated in 6-well plates at a density of 0.5 × 10^5^ cells/ml and cultured for 24 hours before experimentation. Expression of murine PPARγ was knocked down with small interfering RNA (siRNA) duplexes using Oligofectamine (Invitrogen, Carlsbad, CA). The target sequence for PPARγ mRNA was: 5′-AAUAUGACCUGAAGCUCCAAGAAUAAG-3′. Non-targeting siRNA pool (D-001206-13-05; Dharmacon, Fisher Scientific, Pittsburgh, PA) was used as a negative control. Cells were transfected with 1 μg of siRNA in reduced serum medium (OPTI-MEM-I; Invitrogen, Carlsbad, CA) according to the manufacturer's protocol and harvested 72 hours post transfection. Total RNA and protein were extracted and analyzed, respectively.

### Migration assay

Cells (1.0×10^6^ cells/ml) in serum-free medium were added to the top chamber of 24-well transwell plates (8 mm pore size; Corning Star, Cambridge, MA) and 600 μl of complete medium with 10% FBS into the bottom chamber. The assembled chamber was incubated at 37°C in a humidified, 5% CO_2_ cell culture incubator for 24 hours and fixed with 10% formalin and stained with crystal violet (Sigma-Aldrich, St. Louis., MO) for visualization.

### Wound healing assay

Cells (2.0×10^5^ cells/well) were plated in 6-well plates and grown to 80% confluence. The individual wells were wounded by scratching with a pipette tip and incubated with medium containing no FBS to various time points. Wells were photographed under phase-contrast microscopy (×10).

### Animal studies

The study protocols conform to the Guide for the Care and Use of Laboratory Animals published by the US National Institutes of Health (NIH Publication No. 85-23, revised 1996) and was approved by the Animal Ethics Committee at Nanfang Hospital, Southern Medical University, Guangzhou, China. Male Sprague Dawley rats (6-8 weeks of age) were kept in the Animal Center of Nanfang Hospital according to the policy of the Committee for Animal Usage. Diabetes was induced according to the protocol described previously [26, 27]. Briefly, rats were given intraperitoneally a single injection of either streptozotocin (65mg/kg; S0130; Sigma-Aldrich, MO) diluted in 0.1 M citrate buffer pH 4.5 (diabetic) or citrate buffer (non-diabetic). Plasma glucose concentrations were determined using the glucose oxidase method on a glucose analyzer (Accu-check Advantage; Roche, Mississauga, ON) three days after the injection. Rats with a glucose level over 16.7 mmol/L were considered diabetic and thus included in the study. Plasma glucose level was measured once every week. To investigate the effect of CDK5 inhibition on renal tubulointerstitial fibrosis, roscovitine (25 mg/kg; R7772; Sigma-Aldrich, St. Louis., MO) was injected peritoneally to diabetic rats every day till sacrifice. DMSO was included as controls.

Four groups with six to ten rats each were studied: normal rats treated with DMSO (NC-D), normal rats treated with roscovitine (NC-R), diabetic rats treated with DMSO (DM-D) and diabetic rats treated with roscovitine (DM-R). No adverse or toxic effects were observed. Blood glucose level was measured every week. The treatment continued until the rats were euthanized. Twelve weeks after the streptozotocin injection, blood was drawn from the tail vein and plasma samples were prepared for analyzing blood urea nitrogen (BUN), creatinine, β2-microglobulin and glucose level. At 12 weeks after the induction of diabetes, rats were anesthetized with pentobarbital sodium (P3761, 30mg/kg, Sigma-Aldrich, St. Louis., MO). Left kidneys were obtained and fixed in 10% formalin in phosphate buffered saline (PBS) for 24 hours and embedded in paraffin for histological analysis. The right kidney was snap-frozen and stored at −80°C for further analysis.

### Laser capture microdissection (LCM)

Frozen kidney tissues from normal control and diabetic rats were cut at 8 μm thickness and renal tubules and interstitium were microdissected using the PALM MicroBeam LCM system (Zeiss, Germany) according to the manufacturer's instructions.

### Quantitative real-time reverse transcription-pcr analysis

Total RNA from NRK-52E cells and microdissected renal tubules were extracted using TRIzol reagent (MRC, Cincinnati, OH). First strand cDNA was synthesized using 2 μg of total RNA treated with Moloney murine leukemia virus reverse transcriptase (Promega, Madison, WI) according to the manufacturer's instructions. Quantitative real-time reverse transcription-PCR (RT-PCR) analysis was performed in triplicate with Power PCR SYBR Green Master Mix (Applied Biosystems, Carlsbad, CA) using the ABI PRISM 7500 FAST Real-TIME PCR System (Applied Biosystems, Carlsbad, CA) with results normalized to β-actin expression. The ΔΔCT method was used to calculate relative expression. Primer sequences used in RT-PCR are shown in Table 3. All experiments were performed in triplicate.

**Table 3:** Primer sets used in real time RT-PCR

Genes (rat)	Forward Primer	Reverse Primer
*CDK5*	5′-AAGGCACCTACGGAACTGTG-3′	5′-CCCTCATCGTCATCGTCCAG-3′
*P35*	5′-CCAGCTATCGAAAGGCCACA-3′	5′-CCGCTTCAGGTTCTTGTCCT-3′
*ERK1/2*	5′-CATTGTCTCACTGTGTTGCCA-3′	5′-CCAGGAAAGTCAGAAGGCACT-3′
*PPARγ*	5′-CGCAGCCTCAGCCAAGAC-3′	5′-TGGGGAGAGAGGACAGATGG-3′
*E-cadherin*	5′-CCACCAGATGACGATACCCG-3′	5′-GCTTCAGAACCACTCCCCTC-3′
*Vimentin*	5′-TGAGATCGCCACCTACAGGA-3′	5′-GAGTGGGTGTCAACCAGAGG-3′
*Collagen IV*	5′-CCAAGGGAACCAGAGGCTTT-3′	5′-GTGCATCATAACATTTTACTGGACC-3′
*β-actin*	5′-ATGATGATATCGCCGCGCTC-3′	5′-TCGATGGGGTACTTCAGGGT-3′

### Western blot analysis

Lysates from the cells and microdissected renal tubules from each experimental group were separated in parallel on two 10% denaturing sodium dodecyl sulfate-polyacrylamide gels, transferred onto nitrocellulose membranes, blocked with 5% nonfat milk in 0.1% tris buffered saline with Tween-20 (TBST), and probed using antibodies to mouse monoclonal anti-CDK5 antibody (1:100, ab115812, Abcam, Cambridge), rabbit polyclonal anti-p35 antibody (1:100, ab64960, Abcam, Cambridge), rabbit polyclonal anti-ERK1+ERK2 antibody (1:100, ab17942, Abcam, Cambridge), rabbit polyclonal anti-ERK1 (phospho Y204)+ERK2 (phospho Y187) antibody (1:100, ab47339, Abcam, Cambridge), rabbit polyclonal anti-PPAR gamma antibody (1:100, ab19481, Abcam, Cambridge), rabbit polyclonal anti-PPAR gamma (phospho S112) antibody (1:100, Santa Cruz Biotechnology, Santa Cruz, CA), mouse anti-E-cadherin antibody (1:100, ab76055, Abcam, Cambridge), mouse monoclonal vimentin (D21H3) antibody (1:100, #5741, cell signaling technology, MA), and rabbit polyclonal anti-Collagen IV antibody (1:100, ab6586, Abcam, Cambridge) at 4°C overnight. After washing, the secondary antibody (horseradish peroxidase-labeled IgG anti-rabbit/mouse antibody, Invitrogen, Cambridge, MA) was used at 1:3000 dilution for 1 hour at room temperature. The supersignal-enhanced chemoluminescent substrate (Pierce Biotechnology, Inc., Rockford, IL) was applied to the probed membrane and exposed for 10 minutes before the protein bands were visualized on radiograph films (Super Rx, Fuji Photo Film, Tokyo).

### Patients and renal biopsy studies

Total eighty-six renal biopsy samples were obtained from type 2 diabetic patients including 17 from the Division of Nephrology in Nanfang Hospital and 69 from the Department of Renal Pathology at King Medical Diagnostics Center in Guangzhou from 2013 to 2014. The inclusion criteria were: 1) diabetic patients with no history of using renal toxic or herbal medicine; 2) the indications for performing the renal biopsy were proteinuria with or without microscopic hematuria and fast drop in renal function; 3) diabetic patients with no complications of other diagnosed renal diseases. The Ethnics Committee from Southern Medical University and King Medical Diagnostics Center specifically approved the use of patient tissue samples in this study and written informed consent was obtained from each patient.

In all specimens, the morphological diagnosis of DN was confirmed by two individual renal pathologists (JG and XB). The patients were divided into early (n=37) and late staged DN (n=49) according to the pathological staging defined as diffuse or nodular type, respectively. Normal human renal tissues (n=5) from distant portions of kidney tumor were used as controls. Serum and urine samples from the DN patients and healthy volunteers were collected. Blood glucose level, BUN, serum creatinine, serum β2-microglobulin, proteinuria, urine albumin to creatinine ratio (UACR), and estimated glomerular filtration rate (eGFR) were analyzed. Patients clinical data including gender, age, history of diabetes mellitus and blood pressure were examined.

### Immunofluorescence and immunohistochemical analysis

NRK52E cells, tissue samples from the patients and rats were labeled with antibodies to CDK5 (1:100), p35 (1:100), ERK1/2 (1:100), pERK1+pERK2 (1:100), PPAR gamma (1:100), pPPAR gamma (1:100), E-cadherin (1:100), Vimentin (1:100) and Collagen IV (1:100). For immunofluorescence staining, Alexa Fluor 594-conjugated goat anti-mouse IgG and Alexa Fluor 488-conjugated goat anti-rabbit IgG (1:1000, Invitrogen, Cambridge, MA) were used for secondary antibodies, nuclei were counterstained with 4,6-diamidino-2-phenylindole (DAPI, Sigma-Aldrich, St. Louis., MO) and coverslipped with aqueous mounting medium (CTS011, BD Bioscience, Minneapolis, MN). For immunohistochemistry, EnVision™ Detection Systems Peroxidase/diaminobenzidine (DAB), Rabbit/Mouse kit (K4065, Dako, Carpinteria, CA) was used. Nuclei were counterstained with hematoxylin and coverslipped with Permount mounting medium (00-4960-56, eBioscience, San Diego, CA).

Samples were evaluated semiquantitatively by systematically selecting without bias twenty fields for analysis. Images were taken with a BX51 light microscope (Olympus, Tokyo) with appropriate filters. Staining intensity was measured using Image J analysis software (Image J 1.44, National Institute of Health). PBS instead of primary antibodies served as a negative control.

### Evaluation of renal tubulointerstitial fibrosis (TIF)

Five-μm thick paraffin sections were cut for Masson's trichrome stain (MTS). Area of TIF was measured using the Image J analysis software (Image J 1.44, National Institute of Health) by evaluating areas of the injured tubules and interstitium and recorded as interstitial injury score [28, 29]. Briefly, assessment of tubulointerstitial injury was performed and a semiquantitative scoring system as follows was used: 0, normal tubulointerstitium; 1, fibrosis less than 25%; 2, fibrosis between 25% and 50%; 3, fibrosis greater than 50% of the observed fields.

### Statistical analysis

Data are presented as mean ± standard deviation (SD) values. Independent–Samples T Test and One-Way ANOVA followed by Student-Newman-Keuls post hoc test were used to test statistical significance between groups. All statistical tests were performed using SPSS 12.0 (SPSS, Inc., Chicago, IL). The significance level was set at 0.05.

## References

[1] Lai JY, Luo J, O'Connor C, Jing X, Nair V, Ju W, Randolph A, Ben-Dov IZ, Matar RN, Briskin D, Zavadil J, Nelson RG, Tuschl T, Brosius FC, Kretzler M, Bitzer M (2015). MicroRNA-21 in Glomerular Injury. Journal of the American Society of Nephrology : JASN.

[2] Miller N, Feng Z, Edens BM, Yang B, Shi H, Sze CC, Hong BT, Su SC, Cantu JA, Topczewski J, Crawford TO, Ko CP, Sumner CJ, Ma L, Ma YC (2015). Non-aggregating tau phosphorylation by cyclin-dependent kinase 5 contributes to motor neuron degeneration in spinal muscular atrophy. The Journal of neuroscience.

[3] Brinkkoetter PT, Wu JS, Ohse T, Krofft RD, Schermer B, Benzing T, Pippin JW, Shankland SJ (2010). p35, the non-cyclin activator of Cdk5, protects podocytes against apoptosis in vitro and in vivo. Kidney international.

[4] Guevara T, Sancho M, Perez-Paya E, Orzaez M (2014). Role of CDK5/cyclin complexes in ischemia-induced death and survival of renal tubular cells. Cell cycle.

[5] Liu W, Zhang Y, Hao J, Liu S, Liu Q, Zhao S, Shi Y, Duan H (2012). Nestin protects mouse podocytes against high glucose-induced apoptosis by a Cdk5-dependent mechanism. Journal of cellular biochemistry.

[6] Steinman RA, Robinson AR, Feghali-Bostwick CA (2012). Antifibrotic effects of roscovitine in normal and scleroderma fibroblasts. PloS one.

[7] Choi JH, Banks AS, Estall JL, Kajimura S, Bostrom P, Laznik D, Ruas JL, Chalmers MJ, Kamenecka TM, Bluher M, Griffin PR, Spiegelman BM (2010). Anti-diabetic drugs inhibit obesity-linked phosphorylation of PPARgamma by Cdk5. Nature.

[8] Banks AS, McAllister FE, Camporez JP, Zushin PJ, Jurczak MJ, Laznik-Bogoslavski D, Shulman GI, Gygi SP, Spiegelman BM (2015). An ERK/Cdk5 axis controls the diabetogenic actions of PPARgamma. Nature.

[9] Li R, Wang Y, Liu Y, Chen Q, Fu W, Wang H, Cai H, Peng W, Zhang X (2013). Curcumin inhibits transforming growth factor-beta1-induced EMT via PPARgamma pathway, not Smad pathway in renal tubular epithelial cells. PloS one.

[10] Lee YJ, Han HJ (2010). Troglitazone ameliorates high glucose-induced EMT and dysfunction of SGLTs through PI3K/Akt, GSK-3beta, Snail1, and beta-catenin in renal proximal tubule cells. American journal of physiology Renal physiology.

[11] Lopes JP, Agostinho P (2011). Cdk5: multitasking between physiological and pathological conditions. Progress in neurobiology.

[12] Griffin SV, Hiromura K, Pippin J, Petermann AT, Blonski MJ, Krofft R, Takahashi S, Kulkarni AB, Shankland SJ (2004). Cyclin-dependent kinase 5 is a regulator of podocyte differentiation, proliferation, and morphology. The American journal of pathology.

[13] Piedrahita D, Hernandez I, Lopez-Tobon A, Fedorov D, Obara B, Manjunath BS, Boudreau RL, Davidson B, Laferla F, Gallego-Gomez JC, Kosik KS, Cardona-Gomez GP (2010). Silencing of CDK5 reduces neurofibrillary tangles in transgenic alzheimer's mice. The Journal of neuroscience.

[14] Brinkkoetter PT, Olivier P, Wu JS, Henderson S, Krofft RD, Pippin JW, Hockenbery D, Roberts JM, Shankland SJ (2009). Cyclin I activates Cdk5 and regulates expression of Bcl-2 and Bcl-XL in postmitotic mouse cells. The Journal of clinical investigation.

[15] Peng HY, Chen GD, Tung KC, Chien YW, Lai CY, Hsieh MC, Chiu CH, Lai CH, Lee SD, Lin TB (2009). Estrogen-dependent facilitation on spinal reflex potentiation involves the Cdk5/ERK1/2/NR2B cascade in anesthetized rats. American journal of physiology Endocrinology and metabolism.

[16] Tassin TC, Benavides DR, Plattner F, Nishi A, Bibb JA (2015). Regulation of ERK Kinase by MEK1 Kinase Inhibition in the Brain. The Journal of biological chemistry.

[17] Janani C, Ranjitha Kumari BD (2014). PPAR gamma gene - A review. Diabetes & metabolic syndrome.

[18] Cho MC, Lee K, Paik SG, Yoon DY (2008). Peroxisome Proliferators-Activated Receptor (PPAR) Modulators and Metabolic Disorders. PPAR research.

[19] Walton RG, Zhu B, Unal R, Spencer M, Sunkara M, Morris AJ, Charnigo R, Katz WS, Daugherty A, Howatt DA, Kern PA, Finlin BS (2015). Increasing Adipocyte Lipoprotein Lipase Improves Glucose Metabolism in High Fat Diet Induced Obesity. The Journal of biological chemistry.

[20] Lu X, Bijli KM, Ramirez A, Murphy TC, Kleinhenz J, Hart CM (2013). Hypoxia downregulates PPARgamma via an ERK1/2-NF-kappaB-Nox4-dependent mechanism in human pulmonary artery smooth muscle cells. Free radical biology & medicine.

[21] Tian C, Ye X, Zhang R, Long J, Ren W, Ding S, Liao D, Jin X, Wu H, Xu S, Ying C (2013). Green tea polyphenols reduced fat deposits in high fat-fed rats via erk1/2-PPARgamma-adiponectin pathway. PloS one.

[22] Burns KA, Vanden Heuvel JP (2007). Modulation of PPAR activity via phosphorylation. Biochimica et biophysica acta.

[23] Arozal W, Watanabe K, Veeraveedu PT, Ma M, Thandavarayan RA, Sukumaran V, Suzuki K, Kodama M, Aizawa Y (2011). Telmisartan prevents the progression of renal injury in daunorubicin rats with the alteration of angiotensin II and endothelin-1 receptor expression associated with its PPAR-gamma agonist actions. Toxicology.

[24] Kanaguchi Y, Suzuki Y, Osaki K, Sugaya T, Horikoshi S, Tomino Y (2011). Protective effects of L-type fatty acid-binding protein (L-FABP) in proximal tubular cells against glomerular injury in anti-GBM antibody-mediated glomerulonephritis. Nephrology, dialysis, transplantation.

[25] Colangelo T, Fucci A, Votino C, Sabatino L, Pancione M, Laudanna C, Binaschi M, Bigioni M, Maggi CA, Parente D, Forte N, Colantuoni V (2013). MicroRNA-130b promotes tumor development and is associated with poor prognosis in colorectal cancer. Neoplasia.

[26] Bai X, Geng J, Li X, Yang F, Tian J (2014). VEGF-A inhibition ameliorates podocyte apoptosis via repression of activating protein 1 in diabetes. American journal of nephrology.

[27] Bai X, Li X, Tian J, Zhou Z (2014). Antiangiogenic treatment diminishes renal injury and dysfunction via regulation of local AKT in early experimental diabetes. PloS one.

[28] Rodrigues T, Matafome P, Santos-Silva D, Sena C, Seica R (2013). Reduction of methylglyoxal-induced glycation by pyridoxamine improves adipose tissue microvascular lesions. Journal of diabetes research.

[29] Bai X, Geng J, Zhou Z, Tian J, Li X (2016). MicroRNA-130b improves renal tubulointerstitial fibrosis via repression of Snail-induced epithelial-mesenchymal transition in diabetic nephropathy. Scientific reports.

